# Branch-Based Centralized Data Collection for Smart Grids Using Wireless Sensor Networks

**DOI:** 10.3390/s150511854

**Published:** 2015-05-21

**Authors:** Kwangsoo Kim, Seong-il Jin

**Affiliations:** 1 UGS Convergence Research Department, ETRI, 208 Gajeong-ro Yuseong-gu, Daejeon 305-700, Korea; E-Mail: enoch@etri.re.kr; 2 Department of Computer Engineering, Chungnam National University, 99 Daehak-ro Yuseong-gu, Daejeon 305-764, Korea

**Keywords:** smart grid, data collection, wireless sensor network, collision avoidance

## Abstract

A smart grid is one of the most important applications in smart cities. In a smart grid, a smart meter acts as a sensor node in a sensor network, and a central device collects power usage from every smart meter. This paper focuses on a centralized data collection problem of how to collect every power usage from every meter without collisions in an environment in which the time synchronization among smart meters is not guaranteed. To solve the problem, we divide a tree that a sensor network constructs into several branches. A conflict-free query schedule is generated based on the branches. Each power usage is collected according to the schedule. The proposed method has important features: shortening query processing time and avoiding collisions between a query and query responses. We evaluate this method using the ns-2 simulator. The experimental results show that this method can achieve both collision avoidance and fast query processing at the same time. The success rate of data collection at a sink node executing this method is 100%. Its running time is about 35 percent faster than that of the round-robin method, and its memory size is reduced to about 10% of that of the depth-first search method.

## 1. Introduction

Nowadays, wireless sensor networks (WSNs) have begun to be used in smart grids, which intend to provide good power quality, reduce power cost and improve the reliability of the power distribution network. In order to achieve these goals, a smart grid is developed as a type of automated meter reading (AMR) or advanced metering infrastructure (AMI) [1–4] to monitor power system parameters, such as power usage, voltage and current remotely. A power usage collection from each smart meter is one of the most important functions, because the collected data are used in various fields. They are used (1) to create a real-time price for customers, (2) to calculate the real-time electricity cost according to the power consumed by each consumer, (3) to settle on the basic charge for exchanging power at the power exchange, (4) to calculate the total power consumption consumed by all customers in a given period and (5) to estimate the maximum power usage and the moment that the peak load will occur. If there are a lot of missing data, they can cause various problems. As the missing data affect the amount due on electricity bills of customers, a lower amount due charged to customers leads to financial loss for a utility company, whereas a higher amount due leads to customer complaints. Missing data also affect the estimation of the time and amount of the peak load. Thus, incorrect estimations can cause outages, which have numerous social costs, such as halting the function and operation of facilities and equipment [5].

**Figure 1:**
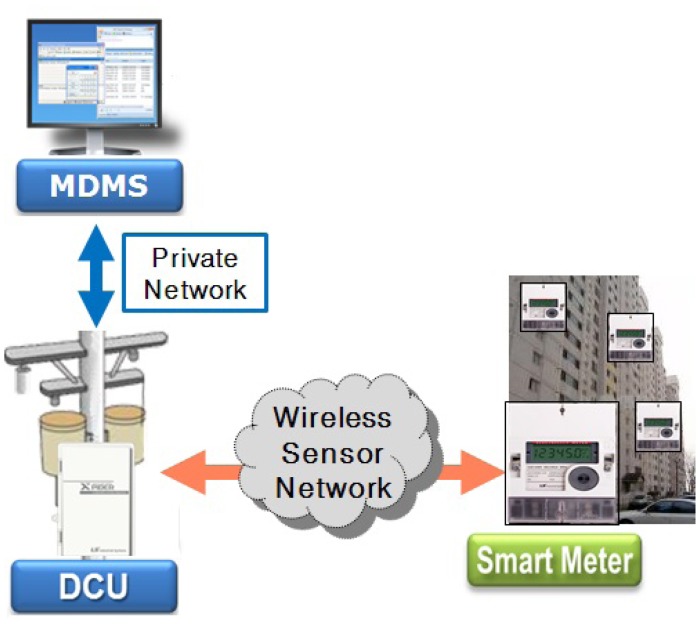
Architecture of the advanced metering infrastructure (AMI) system.

We developed the AMI system with several companies, including Korea Electric Power Company, and installed the system in an apartment complex. It consists of a central server, data collection units (DCUs) and smart meters [6,7]. The system architecture is shown in Figure 1, where MDMS stands for a metering data management server. Smart meters are installed on the corridor walls; DCUs are on the electric polls; and the MDMS is in the utility company. In the system, a WSN with a tree topology has been used between DCUs and smart meters. A smart meter in a smart grid acts as a sensor node, and a DCU acts as a sink node, which is the root of a sensor network. A DCU periodically sends a query to its descendant meters in order to collect the amount of power consumption and status data and transmits these data (e.g., power consumption, power quality, event, monthly data, *etc*.) to the MDMS through a secure private network. The MDMS collects all metering values from DCUs, as well as missing data at midnight. In the system, the company allows each smart meter to have a time difference of about two minutes per month. To collect all data accurately, conventional DCUs developed by electric utilities use the round-robin method, because it is very reliable, can be applied to various network topologies without changes and can collect all data without collisions under an environment in which clock synchronization among smart meters is not guaranteed. However, the method has the critical drawback that it is very slow. Therefore, a utility company has to install many DCUs due to this drawback. This constraint increases the cost to purchase and to install the devices.

To overcome this drawback and reduce costs, we propose an efficient data collection method to collect all data from every smart meter without conflict quickly. The proposed method divides a tree that a sensor network constructs into several branches. Each branch consists of all nodes included in a path from a sink node to a leaf node. We generate a conflict-free query schedule based on the length of each branch to collect all raw data within a given period, one from each of the nodes in a sensor network. The schedule is produced by a sink node that has more resources than other nodes. A sink node sends a query to a scheduled leaf node in a branch, and the scheduled node reports its sensing value to its parent node. Then, each internal node between the sink node and the scheduled node transmits its sensing value, as well as the values received from its child node to the sink node. However, each internal node sends its value generated at the node only once. Therefore, sensing values generated at a node and its descendants in a branch are sent simultaneously to its parent to shorten the data collection time. This method can be used by many utility companies (e.g., power, gas, water, *etc*.). In summary, the contributions of this paper are the following:

- We propose a data collection method based on a branch in a wireless sensor network in which time synchronization among sensor nodes cannot be guaranteed. This method can be used by many utility companies that use wireless sensor networks to collect energy usage data remotely.
- We develop a framework for generating a collision-free schedule based on the length of each branch extracted from a sensor network with a tree topology. The schedule guarantees a minimum memory size to execute the data collection method.
- We propose a query processing scheme, which avoids collisions between disseminated queries and query responses, as well as between query responses and shortens the processing time by sending the sensing values generated at a node and its descendants in a branch to its parent simultaneously.
- We evaluate the performance of data collection methods in terms of the success rate of data collection at a sink node, the memory size required to execute the method at a node and the running time. The experiment results show that our approach is the most suitable method of data collection in a smart grid using a wireless sensor network.

The remainder of the paper is organized as follows. Section 2 presents the related work and compares our approach to existing work. Section 3 describes the problem addressed in this paper and details the design and analysis of the proposed data collection method. Section 4 describes the simulation environment and reports the results obtained using the ns-2 simulator. Section 5 concludes the paper.

## 2. Related Work

In this section, we briefly review some works on data collection methods in WSNs.

Previous data collection algorithms delivering data generated by each node to a sink node [8–11] are based on the assumption that all sensor nodes are synchronized. By considering the interference among nodes, the sink node makes a transmission plan for all nodes. In the plan, interference nodes are assigned to different transmission orders to avoid conflicts among them. The plan is transmitted to all nodes, and each node transmits its sensing value according to its transmission order. However, clock synchronization is a very difficult problem in wireless sensor networks deployed in the real world. If the clocks among nodes are not synchronized, these methods produce wrong results caused by conflicts among interfering nodes.

A reliable bulk transport protocol for multi-hop wireless sensor networks is addressed by [12]. The protocol, Flush, generates only one flow in a sensor network at a time to avoid interference among nodes. To achieve the goal, it has a sink that generates a transfer schedule for each node, and the sink sends onerequest message to a node at a time, in a round-robin fashion, according to the schedule. However, the performance of Flush will be similar to that of a round-robin method when each node in a sensor network does not send bulk data.

A collection tree protocol (CTP), which provides high delivery reliability, is proposed by [13]. Some nodes that support the protocol advertise themselves as tree roots, and others build trees to them. Each node in a sensor network estimates the link qualities between itself and its neighbors, generates expected transmissions (ETX) from them and maintains minimum cost trees using ETX. As the protocol always selects one route with the lowest ETX value from given valid routes, it achieves high delivery reliability. However, the protocol does not promise a 100% delivery rate due to collision. Especially, there will be many lost data if the data collection and the minimum cost tree construction occur simultaneously.

A routing algorithm based on a depth-first search (DFS) method is proposed by [14]. This method forwards a query message to all nodes in a sensor network. A query message is transmitted from a sink node to the first child node of the sink node and is propagated through a sensor network until a leaf node is found. Then, the message returns to the most recent node that the message has not finished exploring. Therefore, an internal node stores all data generated by its descendent nodes and sends them to its parent when the query message finishes traveling to its descendent nodes. The method can reduce the number of query transmissions generated by the sink node and shorten the query response time. However, an internal node that implements this method needs a lot of memory to store data generated by its descendent nodes. The large amount of memory used by DFS is impractical in a real sensor network.

A spanning tree construction method for data collection is proposed by [15]. The method based on a breadth-first search (BFS) starts at a sink node, visits all nodes in a sensor network and constructs a data collection tree. Each node periodically generates a sensing value and sends it to the sink node over the data collection tree. The data collection tree achieves the minimum number of hops between a node and the sink node. However, a collision will occur when several nodes send their sensing values at the same time; therefore, some of the values will not arrive at the sink node due to a possible collision.

In summary, the previous methods are efficient to send a sensing value generated at a sensor node to a sink node in certain ways. In those methods, a sink node makes a transmission plan for all nodes and sends the plan, including the transmission order or time of each node to all nodes, or it initiates a query traversing all nodes and collecting sensing values. By using the plan or the query, the previous methods can avoid possible conflict between data transmissions. However, we can summarize three drawbacks that those methods have. First, clock synchronization among nodes is assumed. Second, the processing time is slow. Third, a large buffer size is required to store sensing values. The clock synchronization and the large buffer size are impractical in a real sensor network. To overcome these shortcomings, a new data collection method has to avoid collisions, shorten the processing time and use a reasonable buffer size under two conditions: asynchronous clocks among nodes and the data collection query issued by a central data collector.

## 3. Centralized Data Collection

In this section, we describe a problem that is considered in this study and a conflict-free data collection method that we propose to solve the problem.

### 3.1. Problem Statement

The scenario we consider consists of a set of sensor nodes, one of which is assigned as the sink node. The clock synchronization among nodes is not guaranteed. In this scenario, the sink node collects all data generated by every node in a senor network within a given period. The sink node periodically sends a query to a sensor node to collect the raw data generated at the node, and the sensor node that receives the query sends its sensing value to the sink node. In the period, each node generates only one sensing value and transmits the value only once.

We now consider the data collection problem at the sink node. The problem is to find a data collection method to minimize the query processing time when the last datum is received by the sink node. Minimizing the query processing time maximizes the number of sensor nodes from which the sink node can collect data within a period *T*. The method should be conflict-free to collect all data, one from each of the nodes in a sensor network. The method also should use a reasonable memory size to be executed at a real node. We first define some notations.

- *N*: a set of sensor nodes
- *T*: data collection period
- *M_d_*: memory size that a node uses to execute a data collection method
- *M*: maximum memory size of a node
- *x*_1_: query transmission time between two adjacent nodes
- *x*_2_: query processing time at a node
- *x*_3_: response transmission time between two adjacent nodes
- *c_i_*: indication of whether a sensing value generated at the *i*-th node arrives at the sink node:
  $$c_{i}=\{\begin{array}{ll} 1, & \text{if the sink receives a sensing value generated at the}\;i-\text{th node}\\ 0, & \text{otherwise} \end{array}$$
- *d*_1,_*_i_*: hop-count between the *i*-th node and a node that issues a query processed by the *i*-th node. The *d*_1,_*_i_* is defined as follows:
  $$d_{1,i}=\{\begin{array}{ll} d_{2,i,} & \text{if the}\;i-\text{th node processes a query sent by the sink node}\\ 1, & \text{if the}\;i-\text{th node processes a query sent by its parent node}\\ 0, & \text{if the}\;i-\text{th node does not processes any query sent by both its parent and the sink} \end{array}$$
- *d*_2,_*_i_*: depth of the *i*-th node. This indicates a hop-count between the *i*-th node and the sink node to send a sensing value generated at the *i*-th node to the sink node.

The problem is to minimize the query processing time as follows:

(1) $$\sum_{i\in N}\left(d_{1,i}x_{1}+x_{2}+d_{2,i}x_{3}\right)$$

In addition, a data collection method solving the problem has to satisfy the conditions as follows:

(2) $$\sum_{i\in N}\left(d_{1,i}x_{1}+x_{2}+d_{2,i}x_{3}\right)\leq T$$

(3) $$\sum_{i\in N}c_{i}=\left|N\right|$$

(4) $$M_{d}\leq M$$

Equation (1) means the query latency, which indicates the time elapsed between initiating the first query at the sink node and receiving the last sensing value. In the Equation (1), the query and the response transmission time, the level of each node and the query processing time might be determined automatically when a sensor network is installed. Equation (2) indicates the constraint that the data collection procedure should be finished within *T*. To finish within *T*, a data collection method has to shorten both the query dissemination time and the query response time between the sink node and another node. Equation (3) indicates that the sensing value generated at a sensor node must arrive at the sink node exactly once within *T*. To collect all sensing values generated at all nodes, the method has to avoid a conflict among message transmissions. In a sensor network, there are two types of messages. One message includes a query transmitted from the sink node to a sensor node, and the other includes a sensing value transmitted from a node to the sink node. Therefore, the method should avoid three types of collisions. One is a collision between disseminated queries and query responses; another is a collision between query responses; the third is a collision between disseminated queries. Equation (4) indicates that the memory size that a node uses to execute the data collection method should be bounded because a senor node has a processing unit with a limited memory. If the data collection method uses a large amount of memory, the method cannot be executed at a node. By considering those constraints, the objective of this study is to design a conflict-free data collection method that brings a query issued at the sink node to an individual node in a sensor network and a sensing value generated at a node to the sink node. Additionally, a node has to execute the method quickly by using a reasonable memory size.

**Figure 2:**
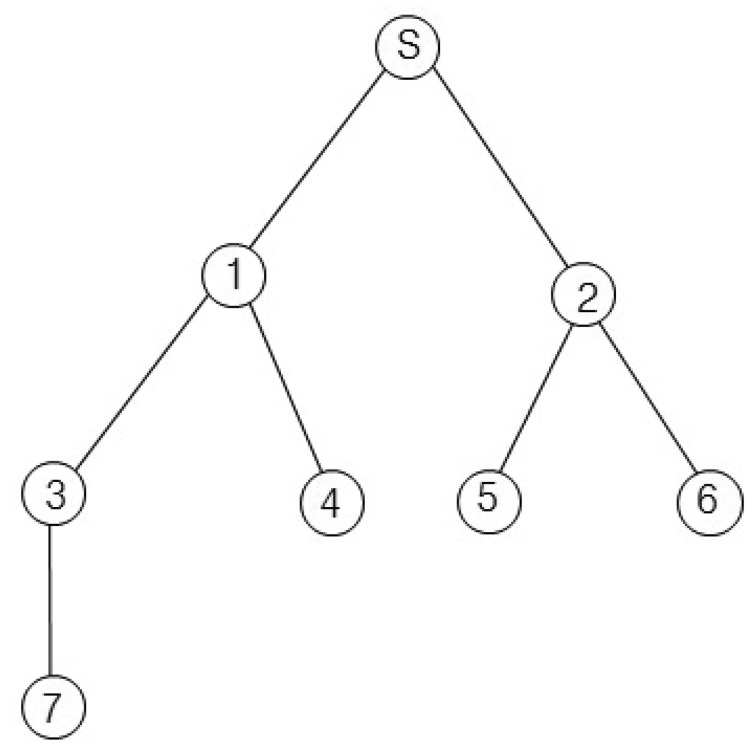
Example of a sensor network.

**Figure 3:**
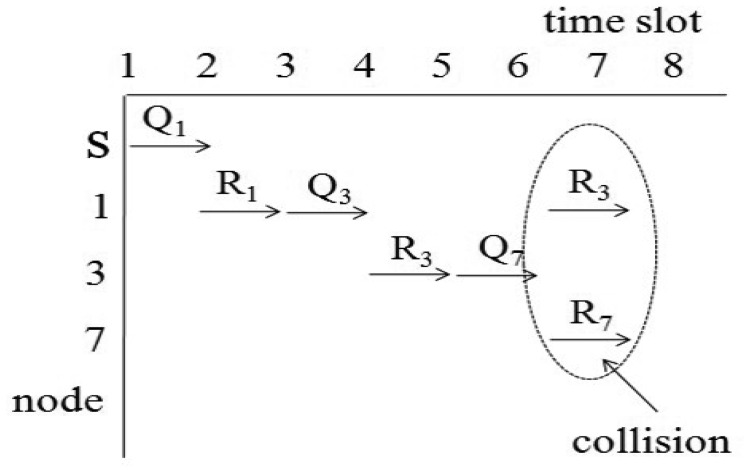
Transmissions in breadth-first search.

For example, we consider the sensor network in Figure 2. The sink node s collects data from seven nodes. Let *x*_1_, *x*_2_ and *x*_3_ of Equation (1) equal one unit of time. If we assume *d*_1,_*_i_* equals *d*_2,_*_i_* for all *i*, then *d*_1,_*_i_* and *d*_2,_*_i_* are one for *i* = {1, 2}, two for *i* = {3, 4, 5, 6} and three for *i* = {7}. Then, the query latency is estimated as 33 units of times. This case is similar to the round-robin method, and the sink node can collect all values without collisions. If we assume *d*_1,_*_i_* equals one for all i and the *d*_2,_*_i_* values for all *i* are the same as those of the first case, the query latency is estimated as 27 unit times. The second case is similar to the breadth-first search method. This method is faster than the round-robin. However, the breadth-first search may cause a collision for a branch consisting of {s, 1, 3, 7}. The collision is shown in Figure 3. In Figure 3, *Q_i_* and *R_i_* denote a query for the *i*-th node and a query response generated at the *i*-th node, respectively. The collision occurs at Node 3 when Node 1 sends *R*_3_ and Node 7 sends *R*_7_ at the same time. Therefore, *R*_7_ will be lost due to the collision.

### 3.2. Conflict-Free Data Collection

**Figure 4:**
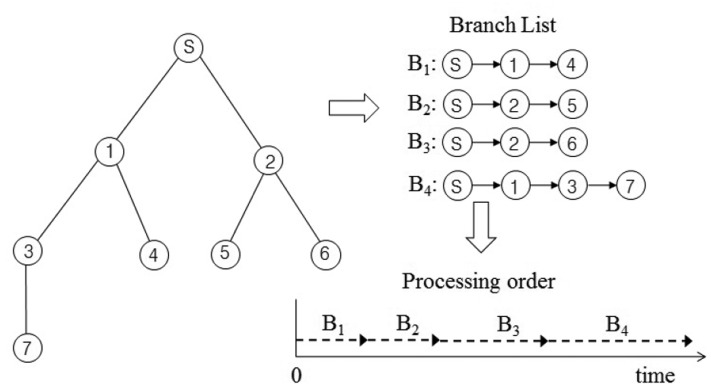
Generating and processing branches.

**Figure 5:**
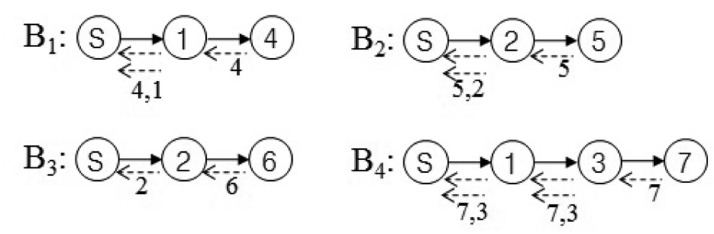
Query and response flows.

In this section, we present our new data collection method to solve the problem discussed in Section 3.1. It is a novel conflict-free query scheduling protocol designed to collect all data, one from each of the nodes in a sensor network. To avoid collisions between transmissions and to shorten data collection time, the data collection scheme uses a branch that is part of the tree of a sensor network. A branch has the following features: (1) a branch consists of all nodes in a path from the sink node to a leaf node; and (2) the union of nodes in all branches equals *N*, such that *B*_1_ ∪ *B*_2_ ∪ ⋯∪ *B_L_* = *N*, where *B_i_* indicates the *i*-th branch. Branches can share the sink node and internal nodes. On the other hand, each branch has one leaf node that is not shared with others. The data collection procedure using branches is shown in Figure 4. The tree is divided into four branches. The branches are processed sequentially to avoid collisions among transmissions. The letter and numbers inside the circles denote node identifiers. The query propagations and query responses in a branch are shown in Figure 5. The solid lines indicate the query propagations from the sink to a leaf node, and the dotted lines indicate the query responses from a node in a branch to the sink. The numbers below the dotted line indicate the node identifiers generating the sensing value. The data collection query is sent to the leaf node in each branch. Node 2 in *B*_2_ sends two sensing values, the value received from Node 5 and its own value, to the sink node. Therefore, there are five message transmissions in *B*_2_. On the other hand, Node 2 in *B*_3_ sends the sensing value received from Node 6, because its own value already was sent while *B*_2_ was being processed.

This data collection scheme consists of two phases: query dissemination and data collection. In the query dissemination phase, the sink node sends a query to the leaf node within a branch. An internal node between the sink and the leaf node forwards the query to its child node as soon as the query arrives. It also prepares its sensing value and waits until its child reports its sensing value. In the data collection phase, the leaf node that receives the query sends its sensing value to its parent. An internal node forwards the data received from its child to its parent as soon as it receives the data and sends its sensing value to its parent only one time, even though it has one or more children. For example, we apply the proposed method to Figure 2 and calculate the query latency using Equation (1). Let *x*_1_, *x*_2_ and *x*_3_ equal one unit of time. Then, *d*_1,_*_i_* is zero for *i* = {1, 2, 3}, two for *i* = {4, 5, 6} and three for *i* = {7}; and *d*_2,_*_i_* is one for *i* = {1, 2}, two for *i* = {3, 4, 5, 6} and three for *i* = {7}. Therefore, the query latency is estimated as 29 units of times. The query latency of the proposed method is faster than that of the round-robin, but slower than that of the breadth-first search. However, the proposed method can collect all values without collisions as the round-robin.

Furthermore, a sensor node needs additional memory to execute the proposed method. The additional memory is needed to store sensing values that a sensor node generates by itself, as well as those it receives from its children. The additional memory size of a sensor node is estimated as follows. Let *M_s_* be the memory size for one sensing value that a sensor node generates and *d_min_* and *d_max_* be the minimum length of a branch and the maximum length of a branch, respectively. Then, the minimum memory size and the maximum memory size required to execute our method are (*M_s_* × *d_min_*) and (*M_s_* × *d_max_*), respectively. Therefore, our method first collects sensing values from the shortest branch to reduce the additional memory size and collects the values in the increasing order of length of each branch.

**Figure 6:**
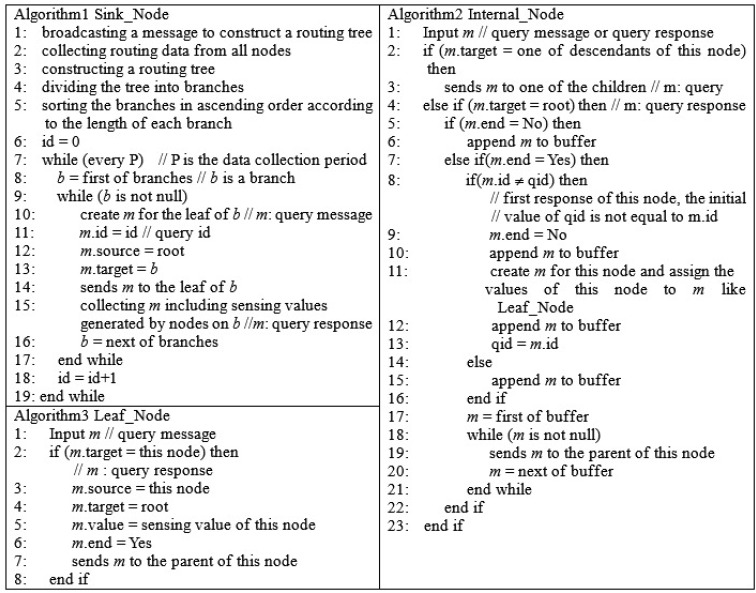
Algorithms for sink, internal and leaf node.

The proposed algorithms are shown in Figure 6. For simplicity, a query message and a query response share the same message consisting of query identifier, source indicator, target indicator, sensing value and end flag. Algorithm 1 describes briefly the processing flow at the sink node. Algorithm 1 can be divided into two parts: tree construction and data collection. In the first part (Line 1 ∼5), a tree is constructed and branches are generated by dividing the tree. The branches are sorted in ascending order according to their length to satisfy Equation (4). In the second part (Line 7 ∼19), this algorithm gives the chance of message transmissions to only one branch to avoid collisions. The algorithm sends a data collection query to the leaf node of a branch and collects the values from the nodes in the branch (Line 14 ∼15). The query identifier is changed after the data collection period is finished (Line 18). Algorithm 2 describes the processing flow at an internal node. The node sends a query message received from its parent to its children if the message's target is one of its descendants (Line 2 ∼3). If the message's target is the sink, the node inserts the message into its buffer until the node receives all messages from its descendants that sent their sensing data (Line 6). When the node receives the last message, it generates the message, including its own values, inserts it into the buffer and sends the messages in the buffer to its parent (Line 7∼22). As the node sends the sensing values without a query whose target is itself, the algorithm shortens the query processing time to satisfy Equation (2). The node sends its values only one time during the period to satisfy Equation (3) (line 8∼13). Algorithm 3 describes the processing flow at a leaf node. When a leaf node receives the data collection query whose target is itself, the node generates the message, including its own values, and sends it to the sink only one time during the period to satisfy Equation (3) (Line 2∼7). In addition, the proposed method easily adapts to topology changes. If the topology changes when a node is inserted into the network or is disconnected, its parent and children reports the event to the sink node. The proposed method reconstructs the tree topology and the branches according to the event. Then, the new branches are used to collect the sensing values.

For simplicity, we assume the transmission time of a message between a sender and a receiver equals one unit of time, and the query processing time at a node is so short that it is negligible. Therefore, the number of transmitted messages is equal to the query latency. However, these assumptions can be relaxed without difficulty.

We can formulate the following properties related to a branch whose length is *d*.

**Property 1.** *(Number of query transmissions) The number of query transmissions from the sink node to a leaf node to collect from a branch equals d.*

**Property 2.** *(Number of response transmissions) The number of response transmissions from each node in a branch to the sink node equals* $\sum_{i=1}^{d}iS_{i}$, *where i indicates the level of a node and S_i_* ∈ {0, 1}.

$$S_{i}=\{\begin{array}{ll} 1, & \text{if a node it the}\;i-\text{th level has its own data to be sent}\\ 0, & \text{otherwise} \end{array}$$

**Property 3.** *(Total number of message transmissions) The total number of message transmissions to collect all sensing values from a branch equals* $d+\sum_{i=1}^{d}iS_{i}$.

By Property 3, we can formulate the minimum query latency and the maximum query latency of a branch as follows.

**Theorem 1.** *(Minimum query latency): The minimum query latency for a branch equals* 2*d*.

The proof is omitted since it is clear from the fact that the minimum query latency occurs when only a leaf node in the branch has its sensing value to be sent to the sink node.

**Theorem 2.** *(Maximum query latency): The maximum query latency for a branch equals* (*d*^2^ + 3*d*)/2.

**Proof.** The maximum query latency occurs when all intermediate nodes and a leaf node in the branch have their sensing values to be sent to the sink node. Let *Q_l_* be the maximum query latency. *Q_l_* can be calculated from Property 3, where *S_i_* = 1 for ∀*i*.

If *d* is an even number,

$$Q_{l}=d+\sum_{i=1}^{d}iS_{i}=d+\left(\left(d+1\right)\times d/2\right)=\left(d^{2}+3d\right)/2$$

If *d* is an odd number,

$$Q_{l}=d+\sum_{i=1}^{d}iS_{i}=d+\left(\left(d+1\right)\times \left(d-1\right)/2+\left(d+1\right)/2\right)=\left(d^{2}+3d\right)/2$$

Therefore, the maximum query latency equals (*d*^2^ + 3*d*)/2.

The maximum query latency is shown in both *B*_1_ and *B*_2_ in Figure 5, because their internal nodes and leaf nodes have a sensing value to be sent. As the length (*d*) of *B*_1_ and *B*_2_ is two, their maximum query latency calculated by Theorem 2 is equal to five units of time. This result is equal to that in Figure 5. The minimum query latency is shown in *B*_3_, because its internal node already sent its sensing value while *B*_2_ was being processed. As the length of *B*_3_ is two, its minimum query latency calculated by Theorem 1 is equal to four units of time. This result is equal to that in Figure 5.

By Theorem 1, we can calculate the minimum query latency of a branch that has shared nodes with another branch. The minimum query latency occurs when the shared nodes already sent their sensing values.

**Corollary 1.** *(Minimum query latency): If a branch shares k nodes with another branch, then its minimum query latency equals* $\frac{d^{2}+3d}{2}-\sum_{i=1}^{k}iH_{i}$, *where k < d; H_i_ indicates whether a node at level i is shared with another branch, and H_i_* ∈ {0,1}.

$$H_{i}=\{\begin{array}{ll} 1, & \text{if a node at the}\;i-\text{th level is shared with another branch}\\ 0, & \text{otherwise} \end{array}$$

Corollary 1 consists of two parts. The former indicates the maximum query latency of a branch, and the latter indicates the sum of query responses of the shared nodes. The minimum query latency related to Corollary 1 is shown in *B*_4_ in Figure 5, because the internal node is shared by *B*_1_, and it already sent its sensing value while *B*_1_ was being processed. As the length of *B*_4_ is three, its maximum query latency calculated by Theorem 2 is equal to nine units of time. As *H_i_* is one for *i* = 1 and *H_i_* is zero for *i* = 2, 3, the sum of query responses of the shared nodes is one. Therefore, its minimum query latency calculated by Corollary 1 is eight units of time. This result is equal to that in Figure 5.

For a sensor network constructing an arbitrary tree, the query latency is estimated as:

(5) $$\sum_{i=1}^{h}\left(i\times L_{i}\right)+\sum_{i=1}^{h}\sum_{j=1}^{N_{i}}\left(1+D_{i,j}\right)$$

where *h* is the height of the tree for a sensor network, *L_i_* is the number of leaf nodes at the *i*-th level, *N_i_* is the number of nodes at the *i*-th level and *D_i,j_* is the number of descendants of the *j*-th node at the *i*-th level.

Equation (5) consists of two parts. The former indicates the sum of query transmissions from the sink to leaf nodes at the *i*-th level, and the latter indicates that of the query responses from nodes at the *i*-th level to the sink.

**Figure 7:**
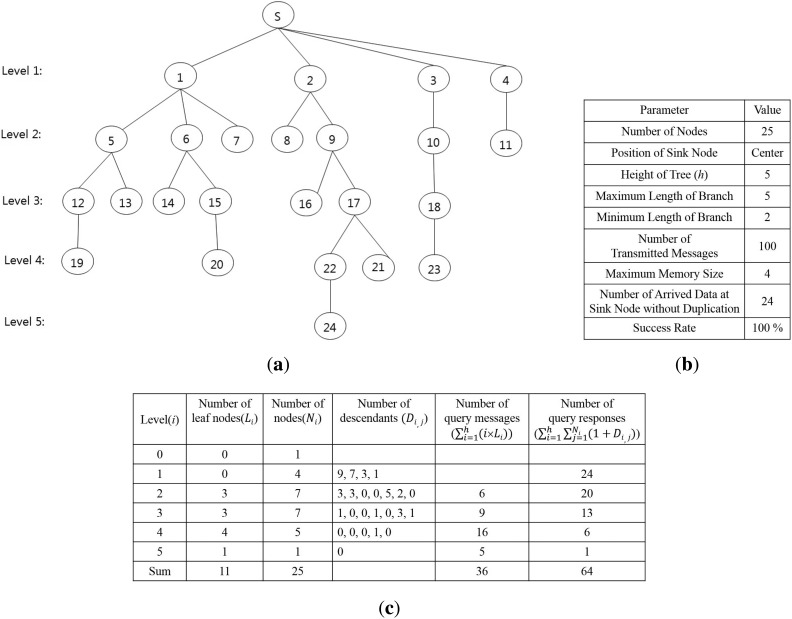
Results of the proposed method and Equation (5). (**a**) Tree structure; (**b**) Execution results of algorithms; (**c**) Number of leaf nodes, descendants and transmitted messages at each level.

To verify the query latency estimated by Equation (5), the result of the equation is compared to that of the proposed method shown in Figure 6. The results are shown in Figure 7. The proposed method generates the tree structure shown in Figure 7a. The property of the tree and the execution results of the proposed method are shown in Figure 7b. The sink node collects 24 data because each node sends its sensing value only one time, like Algorithms 2 and 3. This result also shows that the proposed method satisfies Equation (3) and avoids collisions among message transmissions completely. The maximum memory size to execute the proposed method is four, which is required to process the branch, including {s, 3, 10, 18, 23}. Although the length of the longest branch, including {s, 2, 9, 17, 22, 24}, is five, the memory size for this branch is two, because Nodes 2, 9 and 17 are shared by other branches that were processed earlier than this branch. The query latency of the algorithms is 100 units of time, because the number of transmitted messages is 100. Figure 7c shows the number of leaf nodes at each level and that of descendants of each node, which are used as input values to Equation 5, as well as the results calculated by it. The query latency calculated by Equation (5) is 100 units of time, because the sum of the number of query messages and that of query responses is 100. Therefore, the query latency calculated by Equation (5) is equal to that of the proposed method.

## 4. Performance Evaluation

To study the characteristics of the proposed method, we create a simulation environment based on the ns-2 network simulator [16,17]. It runs on Cygwin, which provides a Linux-like environment under Windows XP. The simulated network is configured as a grid topology in which sensor nodes are uniformly distributed in the network region. As all sensor nodes have the same communication range, the distance between any two nodes is the same.

In a sensor network using the CSkip address scheme, the maximum number of nodes that a sink can have is theoretically 65,535 [18,19]. However, many real applications use fewer than 100 nodes due to collisions. A major electric utility of our country makes 50 to 100 smart meters connect to one DCU. Furthermore, the company allows each smart meter to have a time difference of about two minutes per month. We considered the practical environment and set the number of nodes in the simulation to 121. The count was increased to 169 for a stability test. At the beginning of the simulation, we constructed a routing tree. The node at the center of the grid region was selected as the sink node [20]. The sink node begins the construction of the routing tree by broadcasting a setup message. The maximum number of children of each node in the routing tree is four; the maximum number of route nodes among the children of a node is four; and the height of the routing tree is 11. A random delay time generated by a small jitter was given to each node to generate asynchronous transmission time. It was used to avoid collisions during the tree construction and during the execution of the data collection method. The jitter generates the random delay time in the range of zero to *max_delay*. In this simulation, the *max_delay* was set to 5 ms. A node sends a message after the random delay time expires. The simulation environment is summarized in Table 1, and the property of the branches generated from the sensor network with 121 sensor nodes is summarized in Table 2.

We compared the performance of our algorithm (BR: a data collection method based on a branch) discussed in Section 3.2 with those of several methods with different characteristics in terms of success rate, query latency and memory size. They are broadcasting (BC), breadth first search (BFS), depth first search (DFS) and round robin (RR). The performance of RR is considered as the baseline result. The success rate was calculated by the ratio of the number of arrived messages at the sink node to the number of sensor nodes in the sensor network. The query latency was calculated by the difference between the moment that the sink node sends the first query and the moment that the sink node receives the last response. The memory size indicates the amount of memory that a node uses to store sensing values generated by that node, as well as those received from its descendants while executing each method. The above metrics are related to the constraints discussed in Section 3.1. By considering those constraints, the best method to solve the problem discussed in this paper has to be executed as quickly as possible, use as little memory as possible and collect all sensing values generated by every node.

In each method, the sink node initiates one or more query messages according to the property of each method and collects individual sensing values from all nodes in the sensor network.

### 4.1. Evaluating Success Rate

**Figure 8:**
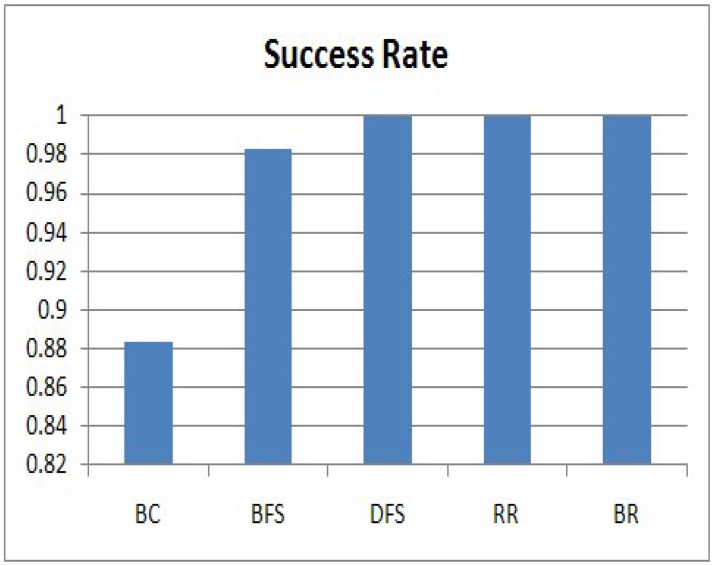
Success rate.

In this section, we evaluate the success rate of the five algorithms, and the results are shown in Figure 8. The success rate is related to Equation (3). The vertical axis expresses the success rate. We can clearly see that DFS, RR and BR achieve a 100% success rate. In the algorithms, individual sensing values from all nodes in the sensor network arrive at the sink node without collisions. However, BC has an 88% success rate and BFS has 98%, because collisions occur among transmissions. For BC, a collision occurs when a query disseminates to each node and a response is forwarded to the sink node. On the other hand, a collision for BFS occurs when a node and its grand-parent send their query responses at the same time because they do not know each other. Therefore, most of the missing responses are generated by nodes at low levels. Considering the success rate, DFS, RR and BR satisfy the requirement of our service scenario, in which the sink node collects all raw data within a given period, one from each of the nodes in a sensor network.

### 4.2. Evaluating Memory Size

**Figure 9:**
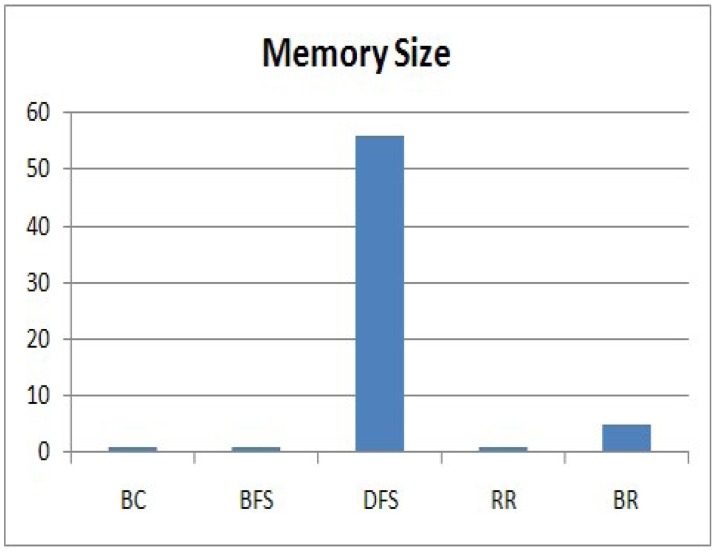
Memory size.

Next, we evaluate the memory size that a node needs to execute the methods. The memory size is related to Equation (4). As all nodes executing each method generate data of the same size, we consider the data generated by a node as one unit size of memory. Even though the memory sizes that individual node require to store data are different, we compare the maximum memory usage among the memory sizes of nodes executing each method. A node executing BC, BFS or RR requires only one unit size of memory, because it generates only one sensing value and forwards the data received from its children as soon as it receives it. For DFS or BR, a leaf node requires one unit size of memory, whereas an internal node requires more memory to store the data received from its descendants. Therefore, we compare the maximum memory usage among the memory sizes that a node needs to execute each method, and the results are shown in Figure 9. The vertical axis expresses the memory size.

We can clearly see that DFS requires a large amount of memory to execute the method at the node. In DFS, an internal node does not send a sensing value received from all of its descendant; rather, it stores the data in its memory to avoid a collision. The node sends the values to its parent after it receives all values from all of its descendants. Therefore, the internal nodes at Level 1 have a lot of memory to store the values received from all of their descendants. However, this is impractical for a sensor node with a limited memory. BR requires a much smaller memory size than DFS. For a branch that has no shared node with another branch, an internal node executing BR requires the maximum *d* unit size of memory, where *d* indicates the length of the branch. For a branch with n shared nodes that have already sent their values, a node requires the maximum (*d* – *n*) unit size of memory. Therefore, the sink node executing BR collects data from a branch in ascending order of its length to reduce the memory size. Even though BR requires a slightly larger memory than RR, it is reasonable. By considering the success rate and the memory size, RR and BR satisfy the requirement of our service scenario, and they are practical methods for a sensor node with a limited memory. The memory size required for BR is about 10% of DFS.

### 4.3. Evaluating Query Latency

**Figure 10:**
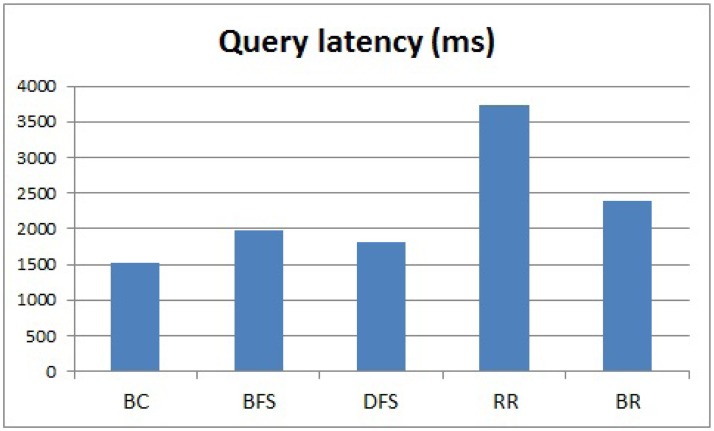
Query latency.

Next, we evaluated the running time of the methods, and the results are shown in Figure 10. The query latency is related to Equation (2). The vertical axis expresses the query latency. We can clearly see that BC is the fastest method because it uses a parallel processing scheme. The other methods considered in this study use a sequential processing scheme. BFS, DFS and BR are faster than RR because they reduce the query transmission time. BFS is slightly slower than DFS because we add an additional message to check whether a query finishes traveling all nodes in a sub-tree of an internal node. However, we cannot use BC and BFS, because their success rates are not 100%, even though BC and BFS are faster than RR. By considering the success rate, memory size and running time, BR is the most suitable method to satisfy all of the requirements of our service scenario for an AMI application using a real sensor network. BR's running time is about 35% faster than RR.

### 4.4. Evaluating Stability

**Figure 11:**
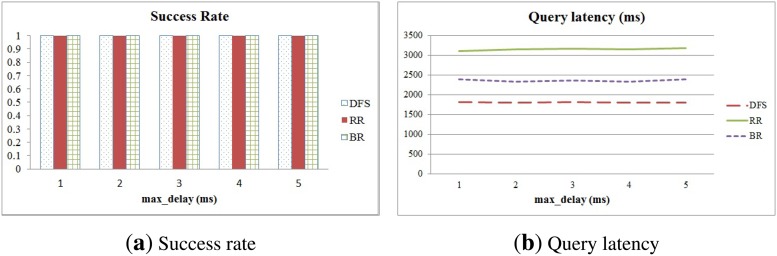
Success rate and query latency in relation to various delay times.

We also evaluated the stability of DFS, RR and BR by means of the impact of the *max_delay* used to generate asynchronous transmission time to avoid collisions and the number of nodes in a sensor network. We varied the *max_delay* from 1 to 5 ms and compared the success rate and query latency of those methods in relation to various delay times. The number of sensor nodes was 121. The results are shown in Figure 11. We can clearly see that the methods are not affected by variation of the delay time, and complete data collection at the sink node is guaranteed.

**Figure 12:**
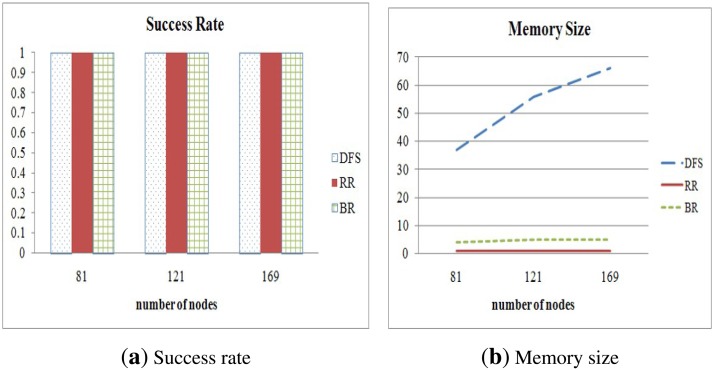
Success rate and memory size in relation to various node counts.

We also varied the number of nodes in a sensor network and compared the success rate and the memory size of those methods according to various node counts. We set 5 ms as the *max_delay*. The results are shown in Figure 12. We can clearly see that the success rate is not affected by variation of the number of nodes in a sensor network. However, the memory size of DFS is affected by the number of sensor nodes, because an internal node executing the method stores all data received from its descendants. On the other hand, the proposed method is only slightly affected by the variation of the number of nodes. Therefore, we conclude that the proposed method is the most suitable to collect all data from nodes in a wireless sensor network, because it can guarantee complete data collection despite environment changes.

## 5. Conclusions

The data collection problem in a smart grid is to collect the power usage from each smart meter without collisions under the condition that the time synchronization among meters is not guaranteed. In the smart grid where a central data collector issues a data collection query and each smart meter sends its power usage to it, the problem is to determine the conflict-free data collection plan during which the query generated at the collector and the power usage generated at each meter arrive at their destinations.

This study proposes a data collection method based on a branch for solving the problem. The proposed method is designed for real applications in a smart grid, which is a practical application area using wireless sensor networks. The proposed method collects all sensing values, one from each of the nodes in a sensor network. A sink node divides a tree into several branches, and each branch consists of all nodes included in a path from the sink node to a leaf node. The sink node periodically sends a query to a leaf node in a branch in ascending order of its length and collects all data generated by nodes in the branch at the same time. This query processing is sequentially executed according to a query scheduling plan. The method has three important features: shortening query processing time, avoiding collisions between messages and using reasonable memory size. In addition, the method easily adapts to topology changes. We compared the proposed method to conventional methods in terms of the success rate of data collection, memory size and running time. The simulation results show that our method is more suitable for a real application than others. The success rate of the proposed method is 100%; the memory size is reasonable; and the running time is about 35% faster than that of the round robin method used by many conventional AMI systems. This result indicates that the proposed method can increase the number of smart meters connected to one central data collector and decrease the cost to purchase and install the central data collectors. Although the proposed method completely avoids collisions in a sensor network rooted at a central data collector, collisions among adjacent nodes included in different sensor networks are not avoided. Therefore, we have to design a global scheduling method to avoid all collisions in the future. In addition, we will study a dynamic scenario with the use of smart sensors.

## Figures and Tables

**Table 1:** Simulation environment.

**Parameter**	**Value**
Topology	Tree
Number of nodes	81, 121, 169
Position of the sink node	Center of the sensor field
Communication range	20 m

**Table 2:** Property of branches for the network with 121 nodes.

**Parameter**	**Value**
Number of branches	49
Minimum length	2
Maximum length	11
Average length	6.9
